# Analysis of risk factors and prediction of prognosis in patients with primary liver cancer undergoing transarterial chemoembolization

**DOI:** 10.3389/fonc.2026.1794553

**Published:** 2026-06-17

**Authors:** Xu-Long Lu, Li-Min Yin

**Affiliations:** Department of Interventional Radiology, Taixing People’s Hospital, Taixing, Jiangsu, China

**Keywords:** cox regression, hepatocellular carcinoma, overall survival, primary liver cancer, prognostic model, progression-free survival, transarterial chemoembolization

## Abstract

**Background:**

Transarterial chemoembolization (TACE) is a key locoregional therapy for unresectable primary liver cancer, yet post-TACE survival remains heterogeneous. We evaluated prognostic determinants and developed a prediction model for overall survival.

**Methods:**

This retrospective cohort study included consecutive adults undergoing index TACE between January 2022 and December 2023. Patients were followed until death or November 30, 2025. Overall survival (OS) and progression-free survival (PFS) were estimated using Kaplan–Meier methods. Candidate predictors were screened using univariable Cox regression and entered into multivariable Cox modeling. A prognostic risk score was derived from model coefficients; discrimination was assessed by Harrell’s concordance index (C-index) and time-dependent receiver operating characteristic (ROC) analyses with area under the ROC curve (AUC), with internal validation.

**Results:**

Among 185 included patients (median follow-up 30.6 months), 85 deaths (45.9%) and 135 progression/death events (73.0%) occurred. Median OS was 28.0 months (12- and 24-month OS: 76.4% and 55.7%), and median PFS was 10.9 months (12- and 24-month PFS: 45.0% and 25.9%). Modified Response Evaluation Criteria in Solid Tumors (mRECIST) responses yielded an objective response rate (ORR) of 48.1% and disease control rate (DCR) of 73.5%. Independent predictors of worse OS included maximum tumor diameter (hazard ratio [HR] 1.137 per 1 cm), portal vein tumor thrombosis (PVTT; HR 2.616), extrahepatic metastasis (HR 1.839), Child–Pugh class B (HR 1.991), international normalized ratio (INR; HR 1.529 per 0.1), and alpha-fetoprotein (AFP; HR 1.946 per 1 log10). The model showed good discrimination (C-index 0.812 training; 0.791 validation) and outperformed established staging/scores.

**Conclusions:**

A parsimonious Cox-based model integrating tumor burden, disease extent, hepatic reserve, coagulation status, and tumor biology provides individualized OS risk stratification after TACE.

## 1 Introduction

Primary liver cancer, predominantly hepatocellular carcinoma (HCC), remains a major cause of cancer-related mortality worldwide and is frequently diagnosed in the setting of chronic liver disease and cirrhosis (1–3). Contemporary international guidelines continue to recommend transarterial chemoembolization (TACE) as a key locoregional treatment for patients with unresectable, liver-confined disease, particularly those with intermediate-stage tumors and preserved hepatic reserve, while emphasizing multidisciplinary decision-making and repeated reassessment of treatment benefit and liver function over time (4–6).

Despite its widespread use, outcomes after TACE remain heterogeneous, reflecting the combined effects of tumor burden and extent, baseline hepatic functional reserve, and host physiological status (7, 8). Recent practice updates have emphasized that the therapeutic benefit of TACE depends not only on procedural technique but also on appropriate patient selection and timely transition to alternative treatment strategies when locoregional control is unlikely or the risk of hepatic decompensation is high (9–11). In parallel, technical consensus statements and procedure-oriented frameworks have been proposed to improve reproducibility and standardize decision pathways for TACE in routine practice, thereby facilitating more consistent outcome assessment across centers (12, 13). Objective evaluation of radiologic response is central to post-TACE management and prognostic stratification (14). Standardized imaging-based systems, including the computed tomography/magnetic resonance imaging Liver Imaging Reporting and Data System treatment response algorithm, have been developed to improve response interpretation and comparability, while quantitative and volumetric response approaches have also been explored to refine survival prediction beyond conventional categorical criteria (15, 16). In addition, liver function metrics, including the albumin-bilirubin (ALBI) grade, have shown prognostic value in TACE-treated cohorts, supporting their integration into risk assessment frameworks alongside tumor-related variables (17, 18).

Given these considerations, there is sustained interest in developing parsimonious prognostic models that integrate routinely available clinical, laboratory, and imaging variables to estimate survival after TACE and facilitate individualized risk communication (19). Recent studies have continued to report and validate predictive tools in TACE-treated populations, while methodological syntheses highlight both the potential and current limitations of artificial intelligence–based approaches for predicting response and outcomes in this setting (20, 21). Against this background, the present study evaluates prognostic determinants and constructs an outcome prediction model for patients with primary liver cancer undergoing TACE within a real-world clinical context.

## 2 Methods

### 2.1 Study design

This retrospective cohort study consecutively enrolled patients with primary liver cancer who underwent TACE at our institution between January 2022 and December 2023. Eligible patients were required to (1) have a diagnosis of primary liver cancer established by histopathology and/or accepted radiologic criteria, (2) receive TACE as an index locoregional treatment during the study period, (3) be aged ≥18 years, and (4) have complete baseline clinical information available for risk-factor evaluation and prognostic modeling, including demographic data, comorbidities, laboratory tests (e.g., liver function and coagulation parameters), and pretreatment imaging assessments. Patients were excluded if they (1) had concurrent or prior malignancies other than primary liver cancer, (2) had received other antitumor therapies before the index TACE that could materially confound post-TACE prognosis (e.g., prior hepatic resection/ablation, radiotherapy, or systemic anticancer therapy), (3) had severe uncontrolled infection or end-stage organ dysfunction precluding standardized assessment (e.g., overt liver failure, severe cardiopulmonary or renal failure), (4) had incomplete key variables or were lost to follow-up immediately after TACE, or (5) were pregnant or lactating. For model development and internal validation, the final analytic cohort of 185 patients was randomly divided into a training cohort (n = 130) and a validation cohort (n = 55) at an approximate ratio of 7:3 using computer-generated random allocation. All procedures involving human participants were conducted in accordance with institutional and national ethical standards and the Declaration of Helsinki. The study protocol was reviewed and approved by the ethics committee of our hospital. Informed consent was obtained from all participants and/or their legal guardians. To ensure confidentiality, all personal identifiers were removed before data analysis.

### 2.2 TACE procedure and peri-procedural management

All patients underwent a standardized pre-procedural evaluation before TACE. Hepatic reserve was assessed using the Child–Pugh classification and the ALBI score. Tumor burden was evaluated on contrast-enhanced computed tomography or magnetic resonance imaging, documenting tumor number and maximal diameter, bilobar involvement, and the presence of macrovascular invasion and/or extrahepatic spread when applicable. Renal function and coagulation status were assessed using serum creatinine (with estimated glomerular filtration rate when available), platelet count, and coagulation parameters, including prothrombin time and international normalized ratio (INR). These assessments were used to guide procedural eligibility and peri-procedural risk mitigation.

TACE was performed by the same experienced interventional radiologist under digital subtraction angiography using a conventional percutaneous arterial approach. After catheterization of the celiac axis and/or superior mesenteric artery, hepatic angiography was conducted to delineate arterial anatomy, identify tumor-feeding arteries, and evaluate portal venous flow when feasible. A microcatheter was advanced into the tumor-feeding branches, and selective or superselective catheterization was preferentially pursued to maximize tumor targeting and reduce non-tumoral hepatic ischemia. A chemotherapy regimen was administered intra-arterially with individualized dosing based on body surface area, tumor burden, and hepatic function. Chemoembolization was subsequently completed using a lipiodol-based emulsion followed by embolic agents consistent with standard practice. The embolization endpoint was defined angiographically by marked reduction of tumor stain and near-stasis of flow within the target vessels while avoiding non-target embolization. Key procedural details, including target vessel selection, administered drugs and approximate doses, lipiodol volume, and embolic materials, were recorded for analysis.

Post-procedural management consisted of supportive care and routine monitoring. Patients received liver-protective therapy and symptomatic treatment for post-embolization syndrome, including antiemetics and analgesics as clinically indicated. Antibiotic prophylaxis or treatment was applied according to institutional practice and individual risk factors. Laboratory monitoring (complete blood count, liver biochemistry, coagulation profile, and renal function) was performed within 24–72 h after TACE, and patients were observed for procedure-related complications, which were documented using a standardized grading approach.

### 2.3 Data collection and follow-up

Clinical data were extracted retrospectively from institutional electronic medical records for all eligible patients undergoing index TACE between January 2022 and December 2023. A standardized case-report form was used to collect baseline variables at, or closest to, the date of the index TACE, including demographic characteristics (age, sex, body mass index), liver disease background (viral hepatitis status, cirrhosis, comorbidities), tumor-related features (maximum tumor diameter, tumor number, portal vein tumor thrombosis (PVTT), extrahepatic metastasis, and Barcelona Clinic Liver Cancer stage), and liver functional reserve indices (Child–Pugh class and albumin–bilirubin grade). Laboratory parameters were retrieved from the most recent pre-procedural tests (typically within 7 days before TACE), including alpha-fetoprotein, albumin, total bilirubin, aminotransferases, platelet count, INR, and derived inflammatory indices when available. Procedural information was abstracted from interventional radiology and anesthesia/nursing records, including TACE modality, embolic materials, chemotherapeutic agents/regimens, and the use of a superselective approach. Data quality was ensured through double-entry verification by two independent investigators, with discrepancies resolved by consensus and reference to source documents; variables with predefined missingness thresholds were handled according to the prespecified statistical plan.

Patients were followed from the date of index TACE until death or the last documented contact. Follow-up information was obtained from outpatient and inpatient records, imaging reports, and telephone contact when necessary. The follow-up cutoff date was November 30, 2025. Survival status and date of death were confirmed through hospital records and, where applicable, linkage to local death registration systems. Tumor assessments were performed using contrast-enhanced CT or MRI at routine post-TACE intervals per institutional practice (typically 4–8 weeks after the procedure and every 8–12 weeks thereafter), and radiologic response was evaluated using modified Response Evaluation Criteria in Solid Tumors (mRECIST). For time-to-event analyses, patients without an event by the cutoff date were censored at the last follow-up, and those lost to follow-up were censored at the last successful contact.

### 2.4 Statistical analysis

All statistical analyses were performed using IBM SPSS Statistics, version 28.0 (IBM Corp., Armonk, NY, USA). Continuous variables were assessed for distributional characteristics and are presented as mean ± standard deviation (SD) for approximately normally distributed data or median (interquartile range [IQR]) for skewed data; categorical variables are summarized as counts and percentages. Between-group comparisons were conducted using the independent-samples t test for normally distributed continuous variables and the Mann–Whitney U test for non-normally distributed variables, while categorical variables were compared using the chi-square test or Fisher’s exact test, as appropriate. Overall survival (OS) and progression-free survival (PFS)were analyzed using the Kaplan–Meier method, and survival curves were compared with the log-rank test. Univariable Cox proportional hazards regression was performed to screen potential predictors of OS, and variables meeting the prespecified selection criterion (P < 0.10) and/or clinical relevance were entered into a multivariable Cox regression model to identify independent prognostic factors. Results are reported as hazard ratios (HRs) with 95% confidence intervals (CIs). Multicollinearity was assessed using variance inflation factors (VIFs). The proportional hazards assumption was evaluated using Schoenfeld residual-based testing; when potential violations were suggested, sensitivity analyses using stratified Cox models were performed.

A prognostic risk score was derived from the final multivariable Cox model coefficients. Model discrimination was evaluated using Harrell’s concordance index (C-index) and time-dependent receiver operating characteristic (ROC) analyses at 1, 2, and 3 years; internal validation was conducted using bootstrap resampling (1, 000 iterations). Comparative discrimination versus established staging/score systems (BCLC stage, Child–Pugh class, ALBI grade, and CLIP score) was assessed using C-index and time-dependent AUC comparisons with paired bootstrap testing. Sensitivity analyses included alternative endpoint definition (OS vs PFS), exclusion of predefined subgroups, and comparison of missing-data strategies (complete-case analysis vs multiple imputation). All tests were two-sided, and a P value < 0.05 was considered statistically significant. Landmark survival analysis was performed to evaluate the association between first post-TACE mRECIST response and subsequent OS.

## 3 Results

### 3.1 Study population and follow-up

Between January 2022 and December 2023, 206 consecutive patients undergoing TACE for primary liver cancer were screened; after applying the predefined eligibility criteria, 21 patients were excluded and 185 patients were included in the final analytic cohort. The primary exclusion reasons were incomplete key baseline clinical/laboratory or imaging data (n = 8), receipt of antitumor therapy prior to the index TACE during the study period (e.g., hepatic resection/ablation, radiotherapy, or systemic therapy) (n = 6), concomitant malignancy or a history of other cancers potentially confounding prognostic assessment (n = 3), failure to meet diagnostic criteria for primary liver cancer on record review (n = 2), and absence of evaluable post-procedural follow-up information (n = 2). Patients were followed from the date of index TACE until death or the last documented contact, and the follow-up cutoff date was November 30, 2025; the median follow-up duration was 30.6 months (interquartile range [IQR], 22.4–39.8 months; range, 1.2–46.9 months). During follow-up, 9 patients (4.9%) were lost to follow-up and were censored at their last successful contact, while patients who remained event-free at the cutoff date were censored at the last follow-up in accordance with standard time-to-event analytic procedures.

### 3.2 Baseline characteristics

Patients were allocated into a training set (n=130) and a validation set (n=55). Overall, the cohort had a mean age of approximately 58 years and was predominantly male, with HBV infection and cirrhosis being common. Tumor burden indicators (maximum tumor diameter, multiplicity, PVTT, and extrahepatic metastasis), staging distributions (BCLC, Child–Pugh, and ALBI), laboratory profiles (AFP, liver enzymes, bilirubin, coagulation, and inflammation indices), and TACE-related characteristics (TACE modality, embolic material, superselective approach, and chemotherapy regimen) were comparable between the training and validation cohorts, with no statistically significant differences detected across baseline variables (all P > 0.05) (**Table 1**).

**Table 1:** Overall baseline balance between the training and validation cohorts.

Variable	Training (n=130)	Validation (n=55)	Test statistic	P value
Demographics				
Age, years	57.36 ± 8.94	60.22 ± 9.16	t = -1.95	0.054
Male sex, n (%)	101 (77.7)	46 (83.6)	χ² = 0.84	0.360
Body mass index, kg/m²	23.98 ± 3.05	24.10 ± 2.96	t = -0.25	0.805
Liver disease background and comorbidities				
HBV infection, n (%)	86 (66.2)	33 (60.0)	χ² = 0.64	0.425
HCV infection, n (%)	8 (6.2)	4 (7.3)	Fisher	0.752
Cirrhosis, n (%)	80 (61.5)	29 (52.7)	χ² = 1.24	0.266
Diabetes mellitus, n (%)	28 (21.5)	9 (16.4)	χ² = 0.65	0.421
Hypertension, n (%)	35 (26.9)	22 (40.0)	χ² = 3.10	0.078
Tumor burden and staging				
Maximum tumor diameter, cm	6.43 ± 2.06	6.17 ± 2.19	t = 0.75	0.457
Multiple tumors, n (%)	72 (55.4)	25 (45.5)	χ² = 1.53	0.216
Portal vein tumor thrombosis, n (%)	34 (26.2)	15 (27.3)	χ² = 0.02	0.875
Extrahepatic metastasis, n (%)	18 (13.8)	7 (12.7)	χ² = 0.04	0.839
BCLC stage, n (%)			χ² = 5.13	0.077
Stage A	30 (23.1)	21 (38.2)		
Stage B	70 (53.8)	21 (38.2)		
Stage C	30 (23.1)	13 (23.6)		
Child–Pugh class, n (%)			χ² = 0.12	0.733
Class A	106 (81.5)	46 (83.6)		
Class B	24 (18.5)	9 (16.4)		
ALBI grade, n (%)			χ² = 0.66	0.719
Grade 1	44 (33.8)	22 (40.0)		
Grade 2	72 (55.4)	28 (50.9)		
Grade 3	14 (10.8)	5 (9.1)		
Laboratory indices				
Alpha-fetoprotein, ng/mL	224.8 (131.3–410.4)	232.4 (154.5–394.2)	U = 3535	0.906
Albumin, g/L	38.57 ± 4.18	37.35 ± 3.96	t = 1.89	0.062
Total bilirubin, μmol/L	16.4 (11.8–21.5)	16.2 (12.4–21.1)	U = 3639	0.849
Alanine aminotransferase, U/L	56.3 (39.3–73.9)	54.9 (38.7–75.0)	U = 3526	0.884
Aspartate aminotransferase, U/L	63.4 (47.0–92.7)	68.2 (49.4–87.7)	U = 3467	0.747
Platelets, ×10^9^/L	148.99 ± 42.78	137.85 ± 48.17	t = 1.49	0.141
International normalized ratio	1.10 ± 0.13	1.12 ± 0.14	t = -1.02	0.310
Neutrophil-to-lymphocyte ratio	2.4 (1.9–3.6)	2.3 (1.8–3.1)	U = 3935	0.280
Platelet-to-lymphocyte ratio	113.9 (84.2–144.6)	128.7 (97.4–168.6)	U = 3066	0.127
TACE-related characteristics				
TACE type, n (%)			χ² = 0.40	0.528
cTACE	114 (87.7)	50 (90.9)		
DEB-TACE	16 (12.3)	5 (9.1)		
Embolic material, n (%)			χ² = 0.53	0.466
Lipiodol + gelatin sponge	95 (73.1)	43 (78.2)		
Lipiodol + PVA particles	35 (26.9)	12 (21.8)		
Superselective catheterization, n (%)	88 (67.7)	40 (72.7)	χ² = 0.46	0.498
Chemotherapy regimen, n (%)			χ² = 0.39	0.823
Epirubicin + platinum	68 (52.3)	31 (56.4)		
Doxorubicin + platinum	44 (33.8)	18 (32.7)		
Other	18 (13.8)	6 (10.9)		

AFP, alpha-fetoprotein; ALB, albumin; ALBI, albumin–bilirubin; ALT, alanine aminotransferase; AST, aspartate aminotransferase; BCLC, Barcelona Clinic Liver Cancer; BMI, body mass index; cTACE, conventional transarterial chemoembolization; DEB-TACE, drug-eluting bead transarterial chemoembolization; HBV, hepatitis B virus; HCV, hepatitis C virus; INR, international normalized ratio; IQR, interquartile range; NLR, neutrophil-to-lymphocyte ratio; PLR, platelet-to-lymphocyte ratio; PLT, platelet count; PVA, polyvinyl alcohol; TACE, transarterial chemoembolization; TBIL, total bilirubin.

### 3.3 Outcomes and survival analysis

A total of 85 deaths were observed during follow-up, corresponding to an OS event rate of 45.9%, whereas 135 patients experienced progression or death, yielding a PFS event rate of 73.0%. The median OS was 28.0 months, with Kaplan–Meier–estimated OS rates of 76.4% at 12 months and 55.7% at 24 months. The median PFS was 10.9 months, and the corresponding PFS rates at 12 and 24 months were 45.0% and 25.9%, respectively. Short-term radiologic response assessed by mRECIST showed CR in 23 patients (12.4%), PR in 66 (35.7%), SD in 47 (25.4%), and PD in 49 (26.5%). Accordingly, the objective response rate (ORR; CR+PR) was 48.1% (89/185) and the disease control rate (DCR; CR+PR+SD) was 73.5% (136/185). Stratified analyses demonstrated significant between-group differences in ORR across clinically relevant risk strata. Patients with AFP <400 ng/mL had a higher ORR than those with AFP ≥400 ng/mL (55.8% vs 33.8%; χ² = 7.31, P = 0.007), whereas DCR was similar between the two AFP strata (73.3% vs 73.8%). Likewise, patients without PVTT achieved a higher ORR compared with those with PVTT (54.6% vs 32.7%; χ² = 6.57, P = 0.010), with comparable DCR values (73.1% vs 74.5%). In addition, the presence of extrahepatic metastasis was associated with a lower ORR relative to patients without extrahepatic metastasis (31.8% vs 53.2%; χ² = 5.31, P = 0.021); DCR was numerically lower in the metastatic subgroup (68.2% vs 75.2%) (**Table 2**).

**Table 2:** Short-term objective response and disease control rates by key clinical strata.

Stratification	Group	n	ORR, n (%)	DCR, n (%)	Test statistic	P value (ORR)
AFP level	AFP <400 ng/mL	120	67 (55.8)	88 (73.3)	χ²=7.31	P < 0.001
	AFP ≥400 ng/mL	65	22 (33.8)	48 (73.8)		
PVTT	No PVTT	130	71 (54.6)	95 (73.1)	χ²=6.57	0.010
	PVTT present	55	18 (32.7)	41 (74.5)		
Extrahepatic metastasis	Absent	141	75 (53.2)	106 (75.2)	χ²=5.31	0.021
	Present	44	14 (31.8)	30 (68.2)		

AFP, alpha-fetoprotein; DCR, disease control rate; mRECIST, modified Response Evaluation Criteria in Solid Tumors; ORR, objective response rate; PVTT, portal vein tumor thrombosis.

### 3.4 Univariable cox regression

Univariable Cox proportional hazards regression was performed to identify candidate predictors of OS. Tumor burden and advanced disease features were associated with increased mortality risk, including larger maximum tumor diameter (per 1-cm increase: HR 1.14, 95% CI 1.03–1.27, P = 0.011), the presence of PVTT (HR 2.70, 95% CI 1.76–4.16, P<0.001), and extrahepatic metastasis (HR 2.40, 95% CI 1.35–4.27, P = 0.003). Markers reflecting impaired hepatic reserve and coagulation dysfunction were also prognostically relevant: Child–Pugh class B (vs A) was associated with substantially worse OS (HR 3.43, 95% CI 2.06–5.71, P<0.001), and higher INR showed an adverse association (per 0.1 increase: HR 1.60, 95% CI 1.35–1.90, P<0.001). In addition, alpha-fetoprotein (AFP) was associated with poorer OS when modeled as a continuous variable on the log10 scale (per 1 log10 increase: HR 2.73, 95% CI 1.38–5.42, P = 0.004), and the association remained directionally consistent when AFP was dichotomized at 400 ng/mL (AFP ≥400 vs <400 ng/mL: HR 1.75, 95% CI 1.10–2.77, P = 0.018). Other baseline factors, including age, sex, body mass index, viral hepatitis status, cirrhosis, and TACE-related procedural characteristics, were not significantly associated with OS in univariable analyses (all P>0.050). Based on a prespecified screening criterion (P<0.100) and clinical plausibility, maximum tumor diameter, AFP, PVTT, extrahepatic metastasis, Child–Pugh class, INR, and BCLC stage (borderline association for stage C vs A, P = 0.071) were selected as candidates for subsequent multivariable modeling (**Table 3**).

**Table 3:** Univariable cox regression for overall survival.

Variable	HR	95% CI	Wald χ²	P value
Age (per 10-year increase)	1.21	0.95–1.54	2.29	0.130
Male sex (yes vs no)	1.05	0.62–1.77	0.03	0.853
Body mass index (per 1 kg/m² increase)	1.02	0.95–1.09	0.25	0.615
HBV infection (yes vs no)	0.76	0.49–1.18	1.49	0.223
Cirrhosis (yes vs no)	0.82	0.53–1.25	0.86	0.353
Diabetes mellitus (yes vs no)	1.06	0.64–1.76	0.05	0.826
Hypertension (yes vs no)	1.22	0.77–1.93	0.79	0.374
Maximum tumor diameter (per 1 cm increase)	1.14	1.03–1.27	6.36	0.011
Multiple tumors (yes vs no)	1.14	0.73–1.78	0.34	0.561
PVTT (present vs absent)	2.70	1.76–4.16	21.62	<0.001
Extrahepatic metastasis (present vs absent)	2.40	1.35–4.27	8.74	0.003
BCLC stage B vs A	1.61	0.90–2.88	2.56	0.110
BCLC stage C vs A	1.81	0.95–3.46	3.27	0.071
Child–Pugh class B vs A	3.43	2.06–5.71	22.02	<0.001
ALBI grade 2 vs 1	1.21	0.74–1.98	0.58	0.446
ALBI grade 3 vs 1	1.61	0.85–3.07	2.12	0.145
AFP (per 1 log10 increase)	2.73	1.38–5.42	8.31	0.004
Albumin (per 1 g/L decrease)	1.04	0.99–1.10	2.62	0.105
Total bilirubin (per 10 μmol/L increase)	1.09	0.92–1.30	0.98	0.322
Platelets (per 50×10^9^/L increase)	0.99	0.78–1.27	0.01	0.916
INR (per 0.1 increase)	1.60	1.35–1.90	29.33	<0.001
DEB-TACE vs cTACE	1.15	0.60–2.23	0.18	0.675
Superselective catheterization (yes vs no)	0.89	0.56–1.41	0.25	0.620
NLR (per 1-unit increase)	0.91	0.70–1.18	0.52	0.471

AFP, alpha-fetoprotein; ALBI, albumin–bilirubin; BCLC, Barcelona Clinic Liver Cancer; cTACE, conventional transarterial chemoembolization; DEB-TACE, drug-eluting bead transarterial chemoembolization; HBV, hepatitis B virus; HR, hazard ratio; INR, international normalized ratio; NLR, neutrophil-to-lymphocyte ratio; OS, overall survival; PVTT, portal vein tumor thrombosis; TACE, transarterial chemoembolization; 95% CI, 95% confidence interval.

### 3.5 Multivariable cox regression

In the multivariable Cox model (**Table 4**), PVTT (adjusted HR 2.616, 95% CI 1.634–4.186, P<0.001), extrahepatic metastasis (adjusted HR 1.839, 95% CI 1.042–3.245, P = 0.036), Child–Pugh class B (adjusted HR 1.991, 95% CI 1.247–3.180, P = 0.004), higher INR (per 0.1 increase: adjusted HR 1.529, 95% CI 1.284–1.822, P<0.001), elevated AFP (per 1 log10 increase: adjusted HR 1.946, 95% CI 1.350–2.804, P<0.001), and larger maximum tumor diameter (per 1 cm increase: adjusted HR 1.137, 95% CI 1.018–1.270, P = 0.023) were independently associated with worse OS after mutual adjustment, whereas age and sex were not significantly associated with OS (both P>0.050). Collinearity diagnostics indicated minimal multicollinearity (all VIF < 2.0). Proportional hazards assessment suggested a potential deviation for Child–Pugh class (P = 0.007), while other covariates did not show evidence of non-proportionality (all P>0.050), and the global test was not significant (P = 0.116). As a sensitivity analysis to address the Child–Pugh PH signal, a stratified Cox model using Child–Pugh class as a stratification factor yielded consistent effect directions and similar magnitudes for the remaining key predictors (e.g., tumor diameter adjusted HR 1.156, P = 0.011; PVTT adjusted HR 2.582, P<0.001; extrahepatic metastasis adjusted HR 1.930, P = 0.024; INR adjusted HR 1.528, P<0.001; AFP adjusted HR 1.997, P<0.001), supporting the robustness of the principal findings.

**Table 4:** Multivariable Cox regression for overall survival.

Variable (coding)	Adjusted HR	95% CI	Wald χ²	P value
Age (per 10-year increase)	1.145	0.882–1.486	1.039	0.308
Male sex (male vs female)	0.975	0.567–1.678	0.008	0.928
Maximum tumor diameter (per 1 cm increase)	1.137	1.018–1.270	5.146	0.023
PVTT (present vs absent)	2.616	1.634–4.186	16.052	<0.001
Extrahepatic metastasis (present vs absent)	1.839	1.042–3.245	4.420	0.036
Child–Pugh class (B vs A)	1.991	1.247–3.180	8.315	0.004
INR (per 0.1 increase)	1.529	1.284–1.822	22.588	<0.001
AFP (per 1 log10 increase)	1.946	1.350–2.804	12.758	<0.001

AFP, alpha-fetoprotein; CI, confidence interval; HR, hazard ratio; INR, international normalized ratio; OS, overall survival; PVTT, portal vein tumor thrombosis.

### 3.6 Discriminative Performance of the Predictive Model

A prognostic model for OS after TACE was constructed using a multivariable Cox proportional hazards framework. The final model included the following independent predictors: maximum tumor diameter (continuous, per 1 cm increase; HR 1.137), PVTT (present vs absent; HR 2.616), extrahepatic metastasis (present vs absent; HR 1.839), Child–Pugh class (B vs A; HR 1.991), INR (per 0.1 increase; HR 1.529), and alpha-fetoprotein (AFP; per 1 log10 increase; HR 1.946). Binary variables were coded as 1 for “present/yes” and 0 for “absent/no, ” and AFP was modeled on the log10 scale. For an individual patient, the linear predictor (risk score) was calculated as: Risk score (LP) = 0.128 × [maximum tumor diameter in cm] + 0.962 × [PVTT (1/0)] + 0.609 × [extrahepatic metastasis (1/0)] + 0.689 × [Child–Pugh class B (1/0)] + 0.425 × [INR per 0.1 unit] + 0.666 × [log10(AFP)]. Accordingly, the patient-specific survival probability at time t can be expressed as: S(t | X) = S0(t) raised to the power of exp (LP), where S0(t) denotes the baseline survival function estimated from the Cox model.

**Figure 1:**
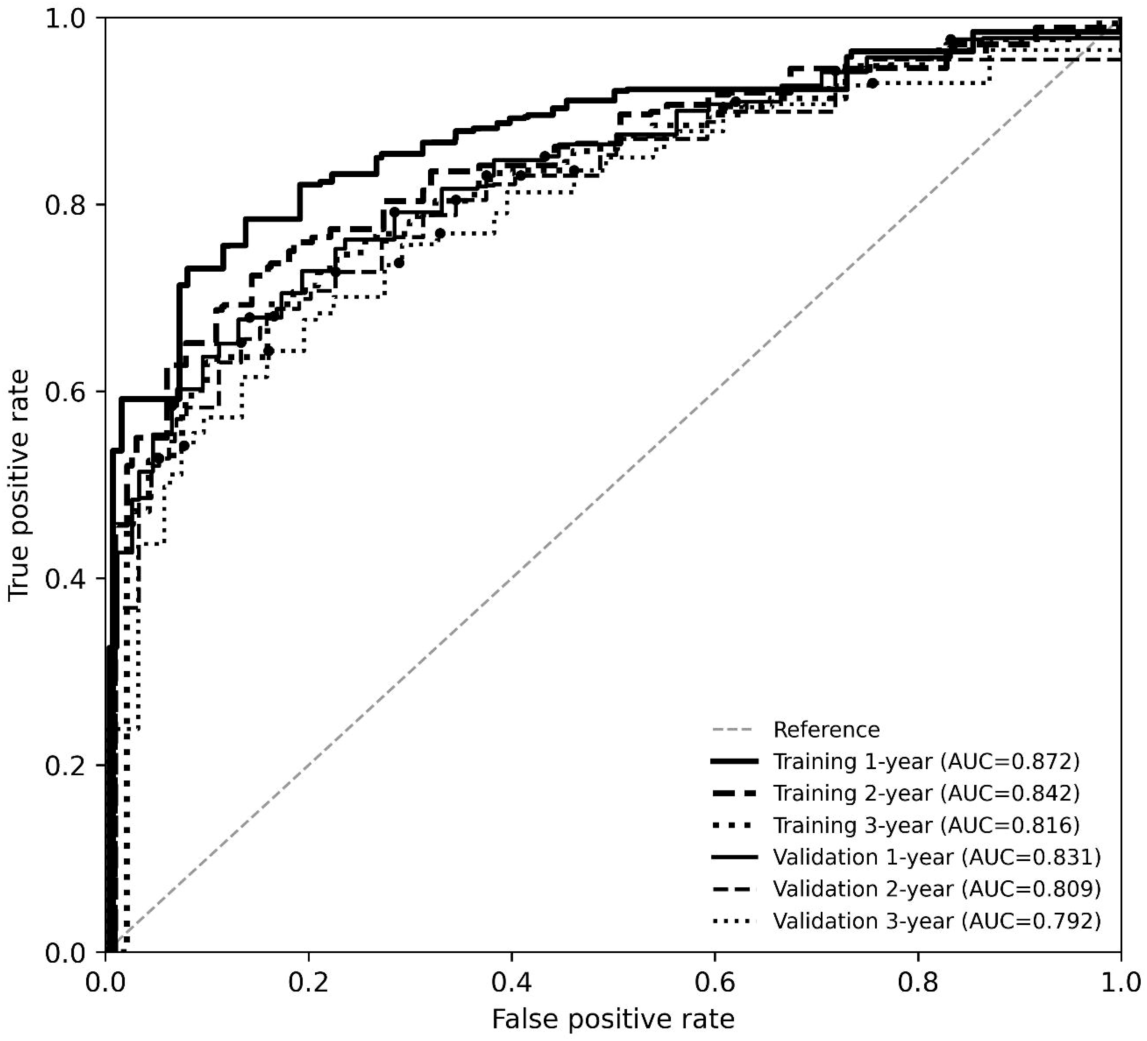
Time-dependent receiver operating characteristic (ROC) curves for prediction of overall survival (OS) at 1, 2, and 3 years in the training and validation cohorts. Training-cohort curves are plotted with thicker lines and validation-cohort curves with thinner lines. The diagonal dashed line represents no-discrimination reference.

Model discrimination was assessed using C-index and time-dependent receiver operating characteristic analysis at 1, 2, and 3 years. The cohort was split into a training set (n=130) and a validation set (n=55), and internal optimism correction was performed using bootstrap resampling (1, 000 iterations). The Cox model incorporating tumor burden (maximum diameter), disease extent (PVTT and extrahepatic metastasis), hepatic reserve (Child–Pugh class), coagulation status (INR), and tumor biology (AFP) demonstrated good discrimination, with a C-index of 0.812 in the training cohort and 0.791 in the validation cohort. Time-dependent AUCs remained stable across 1–3 years (approximately 0.79–0.87), indicating consistent risk separation over clinically relevant time horizons (**Table 5**, **Figure 1**).

**Table 5:** Discrimination performance of the cox model.

Cohort	C-index (95% CI)	AUC at 1 year (95% CI)	AUC at 2 years (95% CI)	AUC at 3 years (95% CI)
Training (n=130)	0.812 (0.758–0.861)	0.872 (0.812–0.926)	0.842 (0.773–0.902)	0.816 (0.736–0.888)
Validation (n=55)	0.791 (0.698–0.875)	0.831 (0.707–0.929)	0.809 (0.662–0.923)	0.792 (0.640–0.910)

AUC, area under the receiver operating characteristic curve; CI, confidence interval.

### 3.7 Comparative performance versus established staging/scores

The Cox prognostic model demonstrated superior discrimination compared with established staging/scores. In the training cohort, the proposed model achieved a higher C-index (0.812) than BCLC stage (0.692), Child–Pugh class (0.636), ALBI grade (0.601), and CLIP score (0.742), with statistically significant differences for all comparisons (P ≤ 0.002). Consistently, time-dependent AUCs at 1, 2, and 3 years were higher for the proposed model (0.872/0.842/0.816) than the corresponding AUCs for BCLC, Child–Pugh, ALBI, and CLIP, with most differences remaining significant. In the validation cohort, the proposed model retained higher C-index (0.791) and higher AUCs across 1–3 years (0.831/0.809/0.792) than BCLC stage and ALBI grade, with significant improvements (P ≤ 0.023), whereas differences versus Child–Pugh class and CLIP score were attenuated and not consistently significant, reflecting greater uncertainty in the smaller validation sample (**Table 6**).

**Table 6:** Comparative discrimination of the Cox model versus established staging/scores.

Model/Score	Training C-index (P)	Validation C-index (P)	Training AUC 1-year (P)	Validation AUC 1-year (P)	Training AUC 2-year (P)	Validation AUC 2-year (P)	Training AUC 3-year (P)	Validation AUC 3-year (P)
Cox model	0.812 (—)	0.791 (—)	0.872 (—)	0.831 (—)	0.842 (—)	0.809 (—)	0.816 (—)	0.792 (—)
BCLC stage	0.692 (<0.001)	0.625 (<0.001)	0.729 (<0.001)	0.612 (0.004)	0.718 (<0.001)	0.630 (0.006)	0.707 (0.012)	0.618 (0.007)
Child–Pugh class	0.636 (<0.001)	0.706 (0.084)	0.671 (<0.001)	0.713 (0.217)	0.652 (<0.001)	0.742 (0.348)	0.603 (<0.001)	0.705 (0.301)
ALBI grade	0.601 (<0.001)	0.649 (0.003)	0.640 (<0.001)	0.642 (0.019)	0.621 (<0.001)	0.661 (0.015)	0.590 (<0.001)	0.659 (0.023)
CLIP score	0.742 (0.002)	0.724 (0.061)	0.812 (0.041)	0.758 (0.476)	0.776 (0.033)	0.736 (0.402)	0.748 (0.048)	0.718 (0.266)

ALBI, Albumin–Bilirubin grade; AUC, area under the receiver operating characteristic curve; BCLC, Barcelona Clinic Liver Cancer staging system; CLIP, Cancer of the Liver Italian Program score.

### 3.8 Sensitivity analyses

Across sensitivity scenarios, the direction of adjusted associations for the core predictors was consistent. When redefining the endpoint from OS to PFS, PVTT, INR, AFP, and maximum tumor diameter remained associated with adverse outcomes, although effect sizes were attenuated relative to OS, consistent with the higher event density for PFS. Excluding patients with extrahepatic metastasis yielded similar effect estimates for tumor burden, PVTT, hepatic reserve, and laboratory predictors. In analyses restricted to Child–Pugh class A, PVTT, INR, and AFP remained associated with OS, whereas the association for extrahepatic metastasis was weaker and did not reach conventional significance in the reduced sample. Handling missing data using complete-case analysis versus multiple imputation produced comparable adjusted HRs and discrimination metrics, suggesting that the primary findings were not materially driven by the missingness strategy (**Supplementary Tables 1**, **2**).

### 3.9 Subgroup analysis

Across stage- and tumor-burden strata, the proposed Cox-based risk score demonstrated broadly consistent discrimination, with C-indices ranging from 0.772 to 0.823 across BCLC stages and from 0.759 to 0.807 across PVTT strata (**Table 7**). Stratification by liver function showed preserved discrimination in Child–Pugh A and B as well as ALBI grade 1–2, while estimates in ALBI grade 3 were less precise due to the smaller sample size and higher event proportion (**Supplementary Table 3**). In stratified adjusted analyses, PVTT, INR, and log10(AFP) retained adverse associations with OS in both Child–Pugh strata, with evidence of effect modification for INR by Child–Pugh class (P for interaction=0.046), whereas PVTT showed no significant interaction with Child–Pugh class (P = 0.214). Additionally, extrahepatic metastasis demonstrated a stronger adverse effect in BCLC stage C compared with earlier stages (P for interaction=0.024), while other tested interactions were not statistically significant (**Supplementary Table 4**).

**Table 7:** Model performance stratified by stage and tumor burden.

Stratification	Subgroup	n (events)	C-index (95% CI)	AUC 1-year (95% CI)	AUC 2-year (95% CI)	AUC 3-year (95% CI)
BCLC stage	A	51 (13)	0.801 (0.720–0.871)	0.846 (0.724–0.944)	0.823 (0.702–0.924)	0.792 (0.656–0.909)
	B	91 (38)	0.823 (0.762–0.878)	0.882 (0.810–0.943)	0.857 (0.781–0.920)	0.829 (0.744–0.902)
	C	43 (34)	0.772 (0.690–0.847)	0.804 (0.668–0.918)	0.781 (0.640–0.900)	0.758 (0.610–0.889)
PVTT	Absent	130 (51)	0.807 (0.752–0.858)	0.869 (0.803–0.929)	0.842 (0.771–0.906)	0.817 (0.732–0.889)
	Present	55 (34)	0.759 (0.678–0.831)	0.786 (0.634–0.914)	0.762 (0.603–0.898)	0.741 (0.575–0.881)
Maximum tumor diameter	<5 cm	48 (14)	0.789 (0.699–0.867)	0.831 (0.700–0.942)	0.812 (0.676–0.929)	0.792 (0.646–0.917)
	≥5 cm	137 (71)	0.809 (0.757–0.858)	0.875 (0.814–0.928)	0.845 (0.778–0.902)	0.818 (0.737–0.890)

AUC, area under the receiver operating characteristic curve; BCLC, Barcelona Clinic Liver Cancer; CI, confidence interval; PVTT, portal vein tumor thrombosis.

### 3.10 First post-TACE mRECIST response and subsequent overall survival

In a landmark survival analysis, post-landmark OS was evaluated according to the first post-TACE radiologic response assessed by mRECIST. Among patients alive at the landmark time, 23 were classified as having complete response (CR), 66 partial response (PR), 47 stable disease (SD), and 49 progressive disease (PD). Patients with CR showed numerically longer post-landmark OS than those with PR, although the difference did not reach statistical significance (median OS, not reached [95% CI, 36.8–NR] vs 33.6 months [95% CI, 27.4–39.8]; 12-month OS, 95.2% vs 85.7%; 24-month OS, 81.4% vs 63.8%; log-rank P = 0.091). In contrast, patients with PR+SD had significantly better post-landmark OS than those with PD (median OS, 28.9 months [95% CI, 24.6–33.2] vs 12.5 months [95% CI, 9.6–15.4]; 12-month OS, 80.6% vs 48.1%; 24-month OS, 55.4% vs 19.7%; log-rank P < 0.001) (**Supplementary Table 5**).

## 4 Discussion

This retrospective study evaluated survival outcomes, short-term radiologic response, and prognostic determinants in patients with primary liver cancer treated with TACE and subsequently developed a Cox-based model for OS. The observed median OS of 28.0 months and median PFS of 10.9 months, together with 12- and 24-month OS rates of 76.4% and 55.7%, indicate that meaningful survival can be achieved in a real-world TACE-treated cohort. At the same time, the high PFS event rate (73.0%) underscores the frequency of early disease progression and the need for refined risk stratification. Short-term response assessed using mRECIST showed an ORR of 48.1% and a DCR of 73.5%, indicating that radiologic tumor control was achieved in a substantial proportion of patients, although progressive disease still occurred in 26.5%. Importantly, ORR varied across clinically relevant strata: elevated alpha-fetoprotein (AFP; ≥400 ng/mL), PVTT, and extrahepatic metastasis were each associated with lower ORR, whereas DCR remained relatively stable across AFP and PVTT strata. This pattern suggests that these adverse features may primarily reduce the likelihood of achieving complete or partial response rather than eliminate the possibility of temporary disease stabilization (21, 22).

Multivariable analysis identified six independent predictors of OS: maximum tumor diameter, PVTT, extrahepatic metastasis, Child–Pugh class B, INR, and log10-transformed AFP. Collectively, these variables reflect tumor burden, disease extent, hepatic functional reserve, coagulation status, and tumor biology. The association between larger tumor diameter and worse OS is consistent with a tumor-burden effect, potentially reflecting greater viable tumor volume, a higher likelihood of satellite lesions, and less complete embolization, particularly when selective catheterization is limited by complex arterial anatomy or multifocal tumor supply. PVTT remained the strongest disease-extent predictor, and its adverse effect is biologically plausible given compromised portal venous flow, increased intrahepatic dissemination, and a heightened risk of hepatic decompensation after ischemic insult (23). Extrahepatic metastasis likewise reflects systemic dissemination and may limit the durability of locoregional control, thereby contributing to both lower ORR and shorter OS. From the host perspective, Child–Pugh class B and elevated INR indicated impaired hepatic reserve and coagulation dysfunction. These factors may limit the intensity of embolization that can be safely delivered, increase susceptibility to post-embolization liver failure, and contribute to mortality independent of tumor progression. AFP, modeled continuously on the log10 scale, remained independently associated with OS, supporting its role as a marker of aggressive tumor biology, microvascular invasion propensity, and treatment resistance. The concordance between predictors of short-term ORR (AFP, PVTT, and metastasis) and predictors of OS suggests that early radiologic response and long-term survival may be influenced by overlapping mechanisms related to tumor biology and disease extent, although hepatic reserve introduces an additional prognostic dimension that may not be fully captured by response categories alone (24).

From a modeling perspective, the derived risk score integrated routinely available clinical variables and demonstrated good discrimination, with C-indices of 0.812 in the training cohort and 0.791 in the validation cohort, as well as stable time-dependent AUCs across 1–3 years. Comparative analyses showed superior discrimination relative to established staging/score systems, including BCLC stage, Child–Pugh class, ALBI grade, and CLIP score, particularly compared with BCLC and ALBI. These findings suggest that a parsimonious multivariable model may provide more refined individual risk stratification in heterogeneous TACE-treated populations than conventional single-domain scoring systems. Sensitivity analyses further supported the robustness of the model across alternative endpoint definitions (OS vs PFS), subgroup exclusions, and different missing-data handling strategies. In subgroup analyses, discrimination remained broadly preserved across BCLC strata and tumor-burden strata, whereas estimates in patients with ALBI grade 3 were less precise, likely reflecting the smaller sample size and higher event burden in this subgroup. Interaction testing suggested that the adverse effect of INR may be stronger in Child–Pugh class B than in Child–Pugh class A, and that extrahepatic metastasis may carry greater prognostic weight in Barcelona Clinic Liver Cancer (BCLC) stage C than in earlier stages. These patterns are clinically plausible: coagulation abnormalities may reflect more advanced hepatic dysfunction in Child–Pugh class B, whereas systemic dissemination may become a dominant determinant of prognosis in advanced-stage disease (4, 25).

Recent guidelines and review articles continue to emphasize that prognosis after TACE is jointly shaped by tumor burden, vascular invasion, extrahepatic spread, and hepatic functional reserve (26–29). Contemporary overviews of HCC management also highlight the central role of locoregional therapy in intermediate-stage disease and the growing need to individualize treatment sequencing and combination strategies according to disease biology and liver reserve (5, 30). In this context, our finding that PVTT and extrahepatic metastasis were independently associated with poorer OS is consistent with the current understanding that macrovascular invasion and systemic dissemination represent more aggressive disease phenotypes, for which the durability of locoregional control is often limited. A 2024 reappraisal of PVTT suggested that selected patients may still derive clinically meaningful benefit from intra-arterial therapies, but outcomes remain highly dependent on careful patient selection and underlying disease characteristics, which is consistent with the persistent adverse association of PVTT in our multivariable model (31, 32).

Liver function has attracted increasing attention beyond static baseline scores. A 2025 analysis of post-TACE ALBI score trajectories showed that dynamic changes in ALBI after TACE were closely associated with outcomes, supporting the broader concept that temporal changes in hepatic reserve contribute meaningfully to prognosis (33, 34). In our study, baseline Child–Pugh class and international normalized ratio (INR) were independent predictors of OS, and the proportional hazards signal observed for Child–Pugh class, together with the consistent findings in stratified sensitivity analyses, is compatible with the notion that liver function may exert a time-varying effect as patients undergo repeated treatment and recover from ischemic injury. In parallel, recent studies have continued to refine TACE-specific prognostic tools that integrate tumor burden and liver function to improve risk stratification. For example, a 2024 report describing a TACE-oriented prognostic score incorporated composite measures of tumor burden together with clinical markers to estimate survival, supporting the ongoing shift from stage-based decision frameworks toward multivariable prediction (35). In addition, the increasing use of discrimination metrics such as the C-index and time-dependent area under the curve (AUC) in recent prognostic modeling studies provides an appropriate methodological context for our performance evaluation, and the observed advantage of our model over Barcelona Clinic Liver Cancer stage and ALBI grade is directionally consistent with the recognized limitations of relatively coarse staging systems in heterogeneous real-world cohorts (36, 37). Collectively, recent evidence supports the importance of macrovascular invasion, systemic dissemination, tumor burden, and hepatic reserve as dominant prognostic domains after intra-arterial therapy, while also supporting multivariable prediction as a useful complement to conventional staging for individualized risk assessment (38, 39).

The identified predictors may support pragmatic risk stratification before TACE. Patients with PVTT, extrahepatic metastasis, elevated AFP, larger tumors, Child–Pugh class B, or increased INR represent a higher-risk phenotype characterized by shorter expected OS and a lower likelihood of objective response. These features may help inform treatment intensity, surveillance frequency, and early consideration of treatment sequencing, including timely transition to systemic therapy when durable locoregional control is unlikely. Consistent reporting of individualized risk estimates, such as predicted 1-, 2-, and 3-year survival probabilities derived from the model, may improve shared decision-making and facilitate standardized baseline stratification in prospective studies. This study integrated survival outcomes, radiologic response, multivariable prognostic modeling, comparative performance against widely used staging and scoring systems, and multiple robustness analyses. Moreover, the selected predictors are routinely available in clinical practice, which may facilitate bedside applicability without the need for specialized imaging biomarkers or omics-based platforms. Model diagnostics showed low multicollinearity (variance inflation factor <2.0), and sensitivity analyses across alternative endpoints and exclusion strategies demonstrated consistent directions and magnitudes of association for the key predictors, thereby strengthening the internal robustness of the findings.

Several limitations should be acknowledged. First, the retrospective design is susceptible to unmeasured confounding, including heterogeneity in embolic intensity, superselective technique feasibility, post-TACE supportive care, and subsequent therapies after progression. Second, the sample size and event counts, particularly within certain subgroups (ALBI grade 3), may reduce precision and can attenuate statistical power for interaction testing, which should be interpreted cautiously. Third, although internal validation suggested stable discrimination, the absence of external validation limits transportability to other centers with different patient selection, etiologic profiles, and practice patterns. Fourth, liver function may change dynamically after TACE; baseline variables may not fully capture longitudinal hepatic reserve, which may contribute to time-varying effects and suggests value in future dynamic modeling using post-treatment trajectories.

## 5 Conclusion

In this retrospective cohort of 185 patients undergoing TACE for primary liver cancer, survival outcomes were moderate (median OS 28.0 months; median PFS 10.9 months). Maximum tumor diameter, PVTT, extrahepatic metastasis, Child–Pugh class B, INR, and AFP independently predicted OS. A Cox-based model integrating these factors demonstrated good discrimination and outperformed conventional staging/score systems, with robustness across sensitivity and subgroup analyses.

## Data Availability

The raw data supporting the conclusions of this article will be made available by the authors, without undue reservation.
