# Supplementary material for: Analysis of risk factors and prediction of prognosis in patients with primary liver cancer undergoing transarterial chemoembolization
Source: Front Oncol. 2026 Jun 17;16:1794553. doi: 10.3389/fonc.2026.1794553 (PMC13318757; doi:10.3389/fonc.2026.1794553)
Supplement: Supplementary file 1 [file Table1.docx]

Supplementary Table 1. Sensitivity analyses for the prognostic model under alternative assumptions

| Scenario | Endpoint | n (events) | C-index (95% CI) | AUC at 1 year (95% CI) |
| --- | --- | --- | --- | --- |
| Primary analysis (final model) | OS | 185 (85) | 0.812 (0.758–0.861) | 0.872 (0.812–0.926) |
| Alternative endpoint definition | PFS | 185 (135) | 0.744 (0.694–0.791) | 0.801 (0.736–0.862) |
| Excluding extrahepatic metastasis | OS | 141 (55) | 0.803 (0.740–0.865) | 0.861 (0.792–0.924) |
| Restricting to Child–Pugh class A | OS | 140 (54) | 0.798 (0.728–0.862) | 0.857 (0.784–0.920) |
| Complete-case analysis* | OS | 166 (76) | 0.809 (0.752–0.862) | 0.868 (0.806–0.924) |
| Multiple imputation* | OS | 185 (85) | 0.811 (0.756–0.860) | 0.871 (0.811–0.926) |

Supplementary Table 2. Adjusted effects of key predictors across scenarios (multivariable Cox models)

| Scenario | Endpoint | Predictor | Adjusted HR | 95% CI | P value |
| --- | --- | --- | --- | --- | --- |
| Primary analysis | OS | Maximum tumor diameter (per 1 cm) | 1.137 | 1.018–1.270 | 0.023 |
|  |  | PVTT (present vs absent) | 2.616 | 1.634–4.186 | <0.001 |
|  |  | Extrahepatic metastasis (present vs absent) | 1.839 | 1.042–3.245 | 0.036 |
|  |  | Child–Pugh class (B vs A) | 1.991 | 1.247–3.180 | 0.004 |
|  |  | INR (per 0.1 increase) | 1.529 | 1.284–1.822 | <0.001 |
|  |  | AFP (per 1 log10 increase) | 1.946 | 1.350–2.804 | <0.001 |
| Alternative endpoint | PFS | Maximum tumor diameter (per 1 cm) | 1.120 | 1.030–1.218 | 0.008 |
|  |  | PVTT (present vs absent) | 1.980 | 1.410–2.780 | <0.001 |
|  |  | Extrahepatic metastasis (present vs absent) | 1.620 | 1.120–2.350 | 0.011 |
|  |  | Child–Pugh class (B vs A) | 1.540 | 1.060–2.230 | 0.024 |
|  |  | INR (per 0.1 increase) | 1.280 | 1.130–1.450 | <0.001 |
|  |  | AFP (per 1 log10 increase) | 1.420 | 1.120–1.790 | 0.004 |
| Excluding metastasis | OS | Maximum tumor diameter (per 1 cm) | 1.150 | 1.020–1.300 | 0.021 |
|  |  | PVTT (present vs absent) | 2.750 | 1.620–4.660 | <0.001 |
|  |  | Child–Pugh class (B vs A) | 2.040 | 1.180–3.520 | 0.011 |
|  |  | INR (per 0.1 increase) | 1.550 | 1.270–1.890 | <0.001 |
|  |  | AFP (per 1 log10 increase) | 1.890 | 1.210–2.950 | 0.005 |
| Child–Pugh A only | OS | Maximum tumor diameter (per 1 cm) | 1.130 | 1.000–1.270 | 0.051 |
|  |  | PVTT (present vs absent) | 2.540 | 1.510–4.290 | <0.001 |
|  |  | Extrahepatic metastasis (present vs absent) | 1.730 | 0.950–3.160 | 0.074 |
|  |  | INR (per 0.1 increase) | 1.470 | 1.200–1.800 | <0.001 |
|  |  | AFP (per 1 log10 increase) | 1.820 | 1.230–2.700 | 0.003 |
| Complete-case | OS | Maximum tumor diameter (per 1 cm) | 1.140 | 1.010–1.290 | 0.032 |
|  |  | PVTT (present vs absent) | 2.580 | 1.550–4.300 | <0.001 |
|  |  | Extrahepatic metastasis (present vs absent) | 1.910 | 1.040–3.500 | 0.037 |
|  |  | Child–Pugh class (B vs A) | 2.050 | 1.230–3.420 | 0.006 |
|  |  | INR (per 0.1 increase) | 1.520 | 1.260–1.840 | <0.001 |
|  |  | AFP (per 1 log10 increase) | 1.930 | 1.300–2.870 | 0.001 |
| Multiple imputation | OS | Maximum tumor diameter (per 1 cm) | 1.130 | 1.010–1.260 | 0.033 |
|  |  | PVTT (present vs absent) | 2.610 | 1.620–4.210 | <0.001 |
|  |  | Extrahepatic metastasis (present vs absent) | 1.820 | 1.030–3.210 | 0.039 |
|  |  | Child–Pugh class (B vs A) | 1.980 | 1.240–3.160 | 0.004 |
|  |  | INR (per 0.1 increase) | 1.510 | 1.270–1.800 | <0.001 |
|  |  | AFP (per 1 log10 increase) | 1.920 | 1.340–2.760 | <0.001 |

AFP, alpha-fetoprotein; AUC, area under the receiver operating characteristic curve; CI, confidence interval; HR, hazard ratio; INR, international normalized ratio; OS, overall survival; PFS, progression-free survival; PVTT, portal vein tumor thrombosis.

Supplementary Table 3. Risk factors and predictive performance stratified by liver function status

| Liver function stratum | n (events) | C-index | Key predictor (coding) | Adjusted HR | 95% CI | P value |
| --- | --- | --- | --- | --- | --- | --- |
| Child–Pugh A | 140 (54) | 0.809 | PVTT (present vs absent) | 2.540 | 1.510–4.290 | <0.001 |
|  |  |  | INR (per 0.1 increase) | 1.470 | 1.200–1.800 | <0.001 |
|  |  |  | AFP (per 1 log10 increase) | 1.820 | 1.230–2.700 | 0.003 |
|  |  |  | Maximum tumor diameter (per 1 cm) | 1.130 | 1.000–1.270 | 0.051 |
| Child–Pugh B | 45 (31) | 0.781 | PVTT (present vs absent) | 2.900 | 1.460–5.770 | 0.002 |
|  |  |  | INR (per 0.1 increase) | 1.780 | 1.340–2.360 | <0.001 |
|  |  |  | AFP (per 1 log10 increase) | 2.200 | 1.260–3.850 | 0.006 |
|  |  |  | Maximum tumor diameter (per 1 cm) | 1.180 | 1.000–1.390 | 0.048 |
| ALBI grade 1–2 | 166 (70) | 0.808 | PVTT (present vs absent) | 2.520 | 1.560–4.080 | <0.001 |
|  |  |  | INR (per 0.1 increase) | 1.510 | 1.260–1.800 | <0.001 |
|  |  |  | AFP (per 1 log10 increase) | 1.890 | 1.300–2.760 | 0.001 |
| ALBI grade 3 | 19 (15) | 0.742 | PVTT (present vs absent) | 2.980 | 1.030–8.630 | 0.044 |
|  |  |  | INR (per 0.1 increase) | 1.920 | 1.270–2.910 | 0.002 |
|  |  |  | AFP (per 1 log10 increase) | 2.480 | 1.070–5.740 | 0.034 |

AFP, alpha-fetoprotein; ALBI, Albumin–Bilirubin grade; CI, confidence interval; HR, hazard ratio; INR, international normalized ratio; OS, overall survival; PVTT, portal vein tumor thrombosis.

Supplementary Table 4. Interaction testing

| Interaction term | Direction (qualitative) | P for interaction |
| --- | --- | --- |
| PVTT × Child–Pugh (B vs A) | PVTT effect similar across Child–Pugh strata | 0.214 |
| INR × Child–Pugh (B vs A) | INR effect stronger in Child–Pugh B | 0.046 |
| AFP × ALBI (grade 3 vs 1–2) | AFP effect numerically higher in ALBI grade 3 | 0.078 |
| Maximum tumor diameter × BCLC (C vs A/B) | Diameter effect comparable across BCLC strata | 0.312 |
| Extrahepatic metastasis × BCLC (C vs A/B) | Metastasis effect stronger in BCLC C | 0.024 |

AFP, alpha-fetoprotein; ALBI, Albumin–Bilirubin grade; BCLC, Barcelona Clinic Liver Cancer; INR, international normalized ratio; OS, overall survival; PVTT, portal vein tumor thrombosis.

Supplementary Table S5. Landmark overall survival according to first post-TACE mRECIST response

| Comparison | Group | n | Median post-landmark OS, months (95% CI) | 12-month OS, % | 24-month OS, % | Log-rank P value |
| --- | --- | --- | --- | --- | --- | --- |
| CR vs PR | CR | 23 | Not reached (36.8–NR) | 95.2 | 81.4 | 0.091 |
|  | PR | 66 | 33.6 (27.4–39.8) | 85.7 | 63.8 |  |
| PR + SD vs PD | PR + SD | 113 | 28.9 (24.6–33.2) | 80.6 | 55.4 | <0.001 |
|  | PD | 49 | 12.5 (9.6–15.4) | 48.1 | 19.7 |  |

Footnote: OS was calculated from the date of the first post-TACE imaging assessment (landmark time). CR, complete response; PR, partial response; SD, stable disease; PD, progressive disease; mRECIST, modified Response Evaluation Criteria in Solid Tumors; NR, not reached.
